# False Bilothorax Secondary to Incarcerated Left Diaphragmatic Hernia With Intestinal Obstruction: A Case Report and Literature Review

**DOI:** 10.7759/cureus.109302

**Published:** 2026-05-20

**Authors:** José A García López, Luis F Manzano Romero, Bryan C Munguia Olvera, Luis F Zorrilla Nuñez, Gerardo E Muñoz-Maldonado

**Affiliations:** 1 General Surgery, Hospital Universitario "Dr. José Eleuterio González", Monterrey, MEX

**Keywords:** bilirubin, bilothorax, intestinal obstruction, pleural effusion, post-traumatic diaphragmatic hernia

## Abstract

Bilothorax, or cholethorax, is defined as the presence of bile within the pleural cavity and represents a very rare entity, classically associated with hepatobiliary injuries, trauma, surgery, or subphrenic abscesses. A pleural-to-serum bilirubin ratio greater than 1 is generally considered suggestive of the diagnosis. Most reported cases occur in the right hemithorax due to the anatomical proximity of the liver, whereas left-sided or bilateral cases are exceptional.

We report the case of a 39-year-old male with a history of prior penetrating thoracic trauma who presented with abdominal pain, nausea, vomiting, and abdominal distension. Computed tomography revealed intestinal obstruction secondary to a left diaphragmatic hernia. Emergency laparotomy was performed, revealing a herniated and ischemic colon, requiring intestinal resection, a Hartmann procedure, and primary closure of the diaphragmatic defect. In the postoperative period, the patient developed a left pleural effusion. Pleural fluid analysis showed an exudate with neutrophilic predominance, numerous erythrocytes, and a total pleural bilirubin level of 3.0 mg/dL, with a concomitant serum bilirubin of 1.9 mg/dL, resulting in a pleural-to-serum ratio of 1.58.

Although the pleural-to-serum bilirubin ratio > 1 meets the classical diagnostic criterion for bilothorax, the clinical presentation, the hemorrhagic appearance of the fluid, and the predominance of indirect bilirubin are more suggestive of a “pseudo-bilothorax” due to intrapleural hemolysis in the context of inflammation and hemorrhage rather than a true biliary effusion. This case highlights the need for cautious interpretation of pleural bilirubin levels in the absence of a clear biliary fistula.

## Introduction

Bilothorax is a rare cause of exudative pleural effusion defined by the presence of bile in the pleural space [1-3]. Most cases reported in the literature are related to hepatobiliary trauma or surgical procedures, such as complicated cholecystectomies, hepatic drainages, or subphrenic abscesses [3,4]. The usual mechanism is the formation of a biliopleural or bilio-peritoneal fistula, allowing bile to pass into the thoracic cavity; less frequently, subdiaphragmatic collections may leak through microdefects in the diaphragm or via lymphatic pathways [1,3].

The most commonly used biochemical criterion for diagnosis is a pleural-to-serum bilirubin ratio greater than 1 [1,4]. However, bilirubin in pleural fluid does not always reflect free bile, as a significant portion may derive from erythrocyte degradation in hemorrhagic or inflammatory effusions [1]. In this sense, the presence of elevated bilirubin in pleural fluid does not necessarily imply the existence of a biliary fistula.

Most cases reported as bilothorax occur on the right side, probably due to the anatomical proximity of the liver [5,6]. Although these hernias may be associated with reactive pleural effusion, it is exceptional for them to be associated with significantly elevated pleural bilirubin in the absence of demonstrable hepatobiliary injury.

The present report describes a case of intestinal obstruction secondary to a left diaphragmatic hernia, complicated by hemorrhagic pleural effusion with an elevated pleural-to-serum bilirubin ratio without evidence of true bile. Its pathophysiological and diagnostic relevance is discussed in light of the available literature [1-7].

## Case presentation

A 39-year-old male presented to the hospital with left lower abdominal pain of seven days’ duration, colicky in nature, associated with nausea, three episodes of vomiting with food content, an absence of bowel movements for three days, and progressive abdominal distension. As relevant history, he reported a gunshot wound to the left hemithorax two years earlier and a stab wound to the left thoracic region eight years earlier, both without surgical repair.

On admission, he was alert and afebrile, with tachycardia and tachypnea. Physical examination revealed a distended abdomen with diffuse tenderness, more pronounced in the left flank; decreased bowel sounds; and no clear signs of peritoneal irritation. Thoracic examination showed preserved lung expansion and symmetrical vesicular breath sounds without significant pathological findings.

Computed tomography revealed a left diaphragmatic defect of approximately 2.1 cm with protrusion of transverse colon loops, greater omentum, and adjacent visceral fat into the left hemithorax, causing marked proximal dilation and findings compatible with mechanical intestinal obstruction due to diaphragmatic herniation. No hepatic injuries or subphrenic collections were documented (Figures 1, 2).

**Figure 1:**
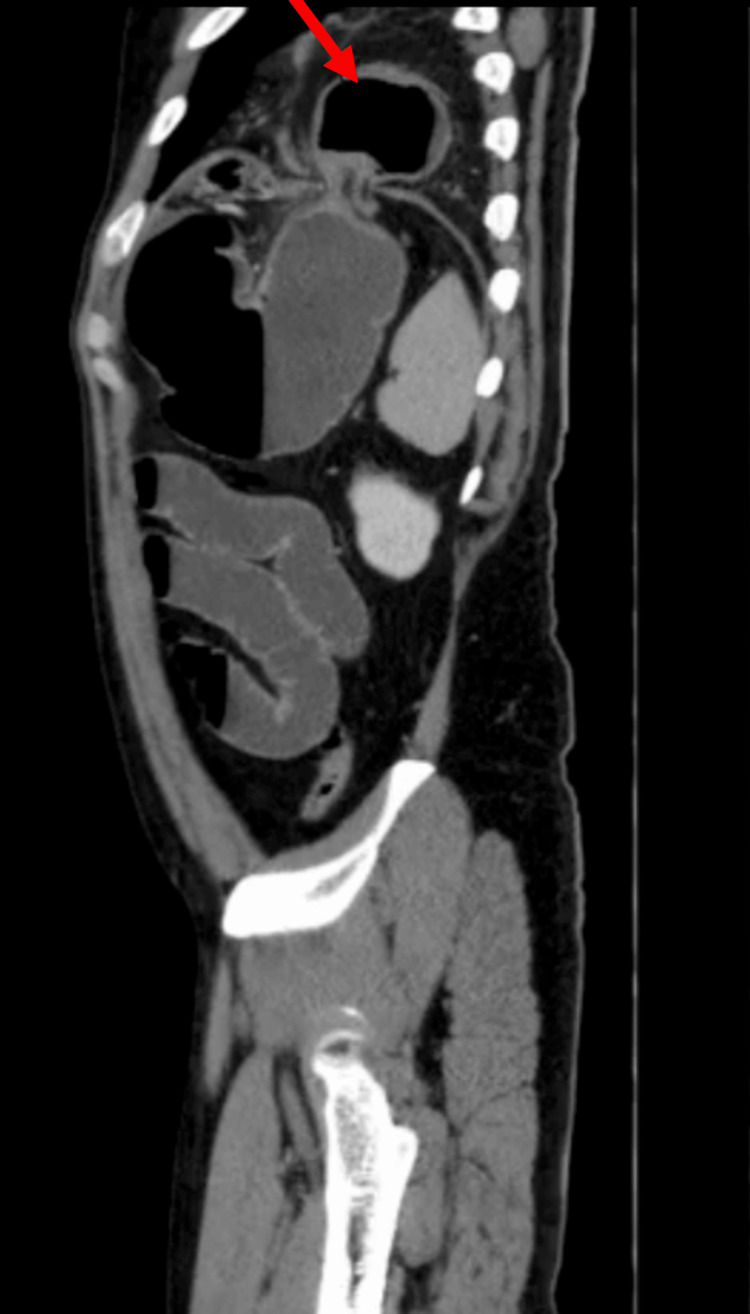
Sagittal reformatted contrast-enhanced computed tomography image through the upper abdomen and lower chest Demonstrating a 2.1 cm defect in the left hemidiaphragm (red arrow), with herniation of a colonic segment into the thoracic cavity.

**Figure 2:**
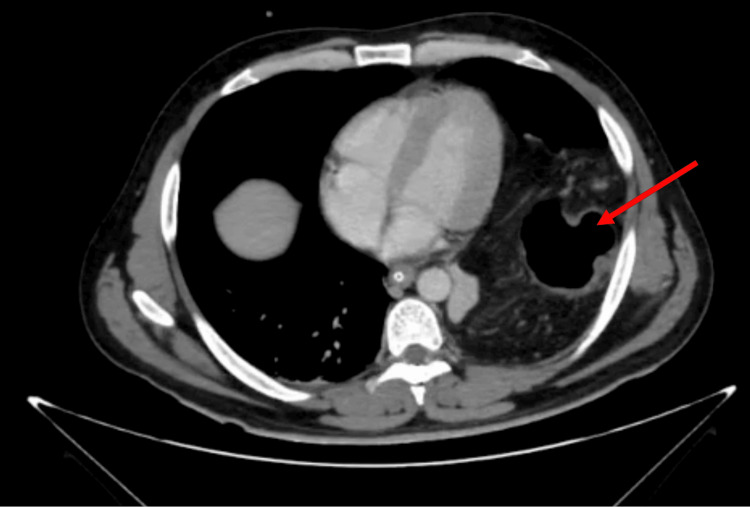
Axial contrast-enhanced computed tomography image through the lower chest Showing a herniated, gas-containing colonic segment with a visible intestinal wall adjacent to the pleura and partially collapsed left lung (red arrow).

Based on the diagnosis of intestinal obstruction secondary to an incarcerated diaphragmatic hernia, emergency laparotomy was performed. Herniation of the transverse colon into the left hemithorax through the diaphragmatic defect was identified, with ischemic compromise of the right hemicolon. Resection of the nonviable cecum and ascending colon was performed, with primary closure of the diaphragmatic defect. A right hemicolectomy, terminal ileostomy, and a long Hartmann’s pouch of the transverse colon were created (Figures 3, 4).

**Figure 3:**
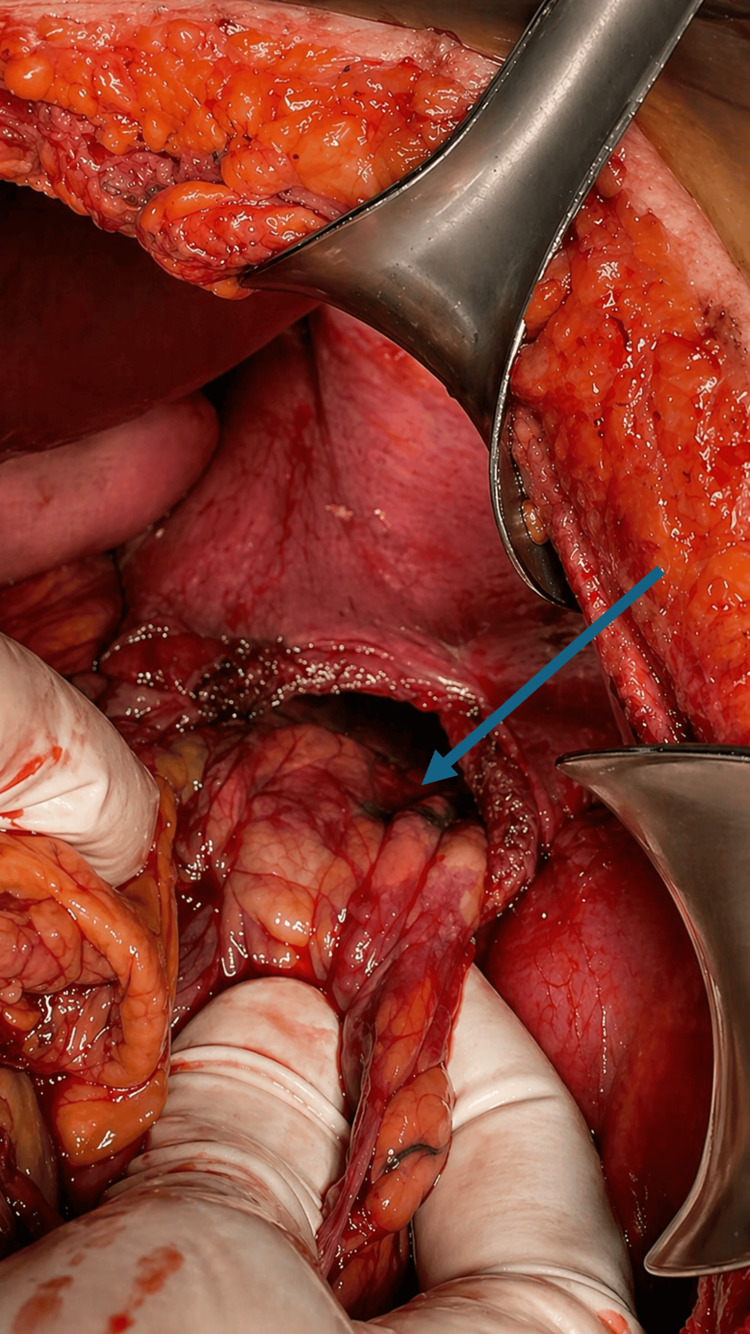
Diaphragmatic hernia defect evidenced intraoperatively Showing an orifice in the abdominal wall with protrusion of intra-abdominal contents through it (blue arrow).

**Figure 4:**
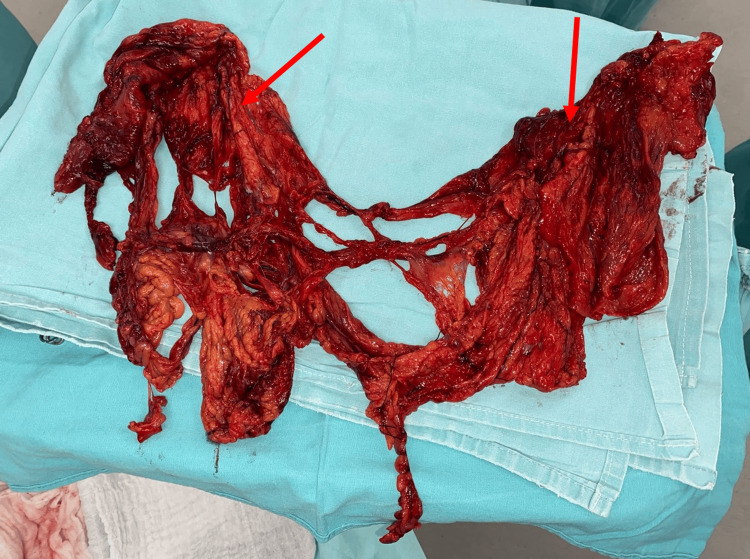
Intraoperative photograph of the herniated contents after surgical reduction An extensive segment of the colon with its associated omentum is visible (red arrows). The tissue shows marked congestion, mesenteric edema, and areas of vascular compromise, findings compatible with strangulation secondary to hernia incarceration.

Twenty-four hours postoperatively, after a left pleural effusion was observed on chest radiography, diagnostic and therapeutic thoracentesis was performed in the left hemithorax, obtaining approximately 200 mL of pleural fluid (Figure 5). Subsequently, a single drainage catheter was placed. 

**Figure 5:**
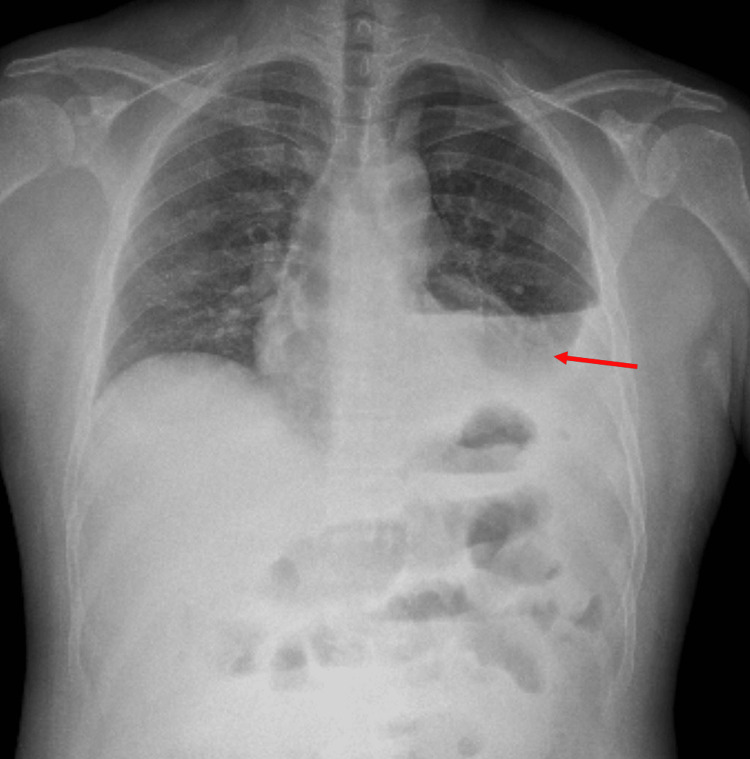
Frontal chest radiograph Demonstrating an air-fluid level within the left pleural space (red arrow).

The obtained fluid was hemorrhagic with a biliary-tinged appearance (Figure 6). Analysis showed 9,750 leukocytes/mm³ (97% polymorphonuclear cells) and 250,000 erythrocytes/mm³, with protein at 2.5 g/dL, lactate dehydrogenase (LDH) at 1,858 U/L, and glucose at 78 mg/dL. Total bilirubin in pleural fluid was 3.0 mg/dL (direct 0.4 mg/dL, indirect 2.6 mg/dL). Simultaneous serum bilirubin was 1.9 mg/dL, yielding a pleural-to-serum bilirubin ratio of 1.58.

**Figure 6:**
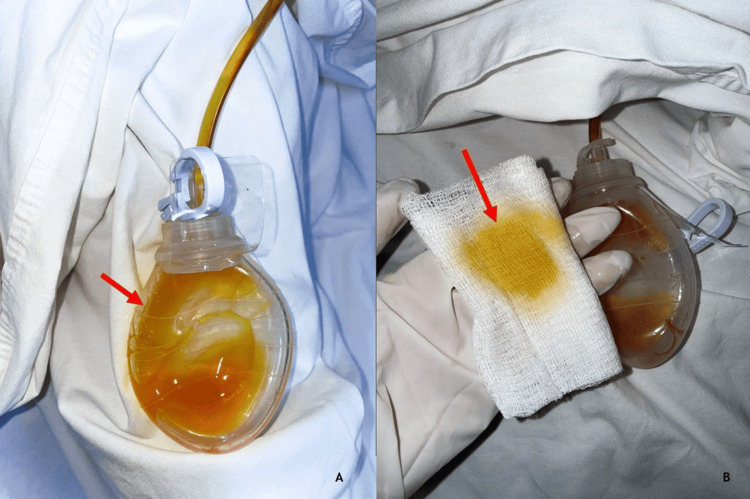
Gross appearance of the pleural fluid obtained by catheter A, B: Demonstrating a hemorrhagic fluid with a distinctive biliary-tinged, dark yellow–brown coloration (red arrows).

During the 24 hours following catheter placement, the patient remained under close monitoring to quantify drainage and to assess for the potential development of an acute abdomen or progression of respiratory insufficiency.

As part of follow-up, a control thoracoabdominal computed tomography scan was performed to rule out thoracoabdominal communication, evaluate the evolution of the pleural effusion, and exclude the presence of a fistula, as well as to identify other possible associated intra-abdominal or intrathoracic complications (Figure 7).

**Figure 7:**
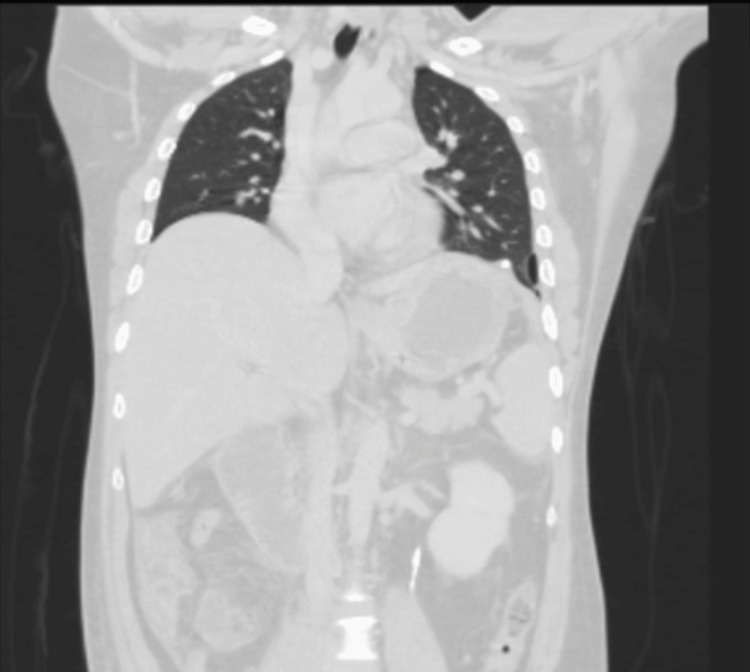
Non-contrast chest CT in lung window Demonstrating no pleural effusion, pneumothorax, or significant atelectatic changes. No associated upper abdominal abnormalities are identified.

## Discussion

This case highlights that a pleural-to-serum bilirubin ratio greater than 1 does not necessarily indicate true bilothorax. Interpretation should integrate the fluid’s color, the anatomical context, bilirubin fractions, and evidence of a possible biliary fistula [1,3,4].

From a pathophysiological standpoint, pleural bilirubin may increase in effusions with a high erythrocyte content due to red blood cell degradation. In this patient, the predominance of indirect bilirubin together with the large number of erythrocytes in the pleural fluid supports this explanation. In other words, the bilirubin detected in the pleural space may represent the end product of local hemolysis, rather than bile originating from the biliary tree.

Some studies have suggested that the pleural-to-serum bilirubin ratio may help distinguish exudates from transudates, but its diagnostic value is not absolute and must be interpreted within a broader clinical context [1]. Furthermore, recent reports of malignant brown pleural effusions have demonstrated markedly elevated pleural bilirubin levels without demonstrable hepatobiliary injury, reinforcing the concept that this index may increase through non-biliary mechanisms [2]. Comparison of key characteristics among reported cases of bilothorax is summarized in Table 1.

**Table 1:** Comparison of key characteristics among reported cases of bilothorax * In Najjar, most cases were in the right hemithorax (note that a left bilithorax is extremely rare) [7]; ** In Vrachliotis, bile was drained with a PF/S < 1 because the serum bilirubin was very high [8]. N/A: not available; AB: antibiotic therapy; PF/S: pleural fluid-to-serum; PMN: polymorphonuclear

Source (year)	Patients (n)	Side	Etiology	Pleural bilirubin (mg/dL)	Serum bilirubin (mg/dL)	PF/S ratio	Liquid appearance	PMN (%)	Red blood cells (/µL)	Management	Result
Najjar et al. (2018) [7]	36	Right*	Hepatobiliary tumors, abscesses, trauma	– (average ~50–100)	– (average ~20–50)	>1 (criterion)	Yellowish green	N/A	N/A	Drainage + correction	↑ Morbidity and mortality
Abid et al. (2025) [3]	1	Right	Gallbladder perforation	1.4	0.3	4.67	Deep green	N/A	N/A	Drainage + AB, surgery	Deceased
Vrachliotis et al. (2022) [8]	1	Right	Percutaneous biliary drainage	12	15	0.8**	Biliary (green)	N/A	N/A	Repositioning + tract embolization	Limited survival
Current case (2026)	1	Left	Post-traumatic diaphragmatic hernia	3	1.9	1.58	Bloody/Biliary (green)	97	250,000	Pleural drainage + AB	Full recovery

Figure 8 illustrates the conceptual value of the pleural fluid-to-serum bilirubin ratio in bilothorax while also showing its limitations when interpreted in isolation. The best available evidence defines bilothorax as bile in the pleural space and confirms the diagnosis when the pleural fluid-to-serum bilirubin ratio exceeds 1.0. In the largest systematic review available, 115 pooled cases yielded a mean ratio of 3.47, and most cases occurred after hepatobiliary surgery/procedures or thoracoabdominal trauma. Earlier diagnostic work likewise supported the > 1.0 threshold, although its sensitivity was only 76.9% when used alone and improved when pleural glycocholic acid was added. These data support the ratio as a strong biochemical marker, but not as a stand-alone diagnostic endpoint. In the present case, a ratio of 1.58 supports bilothorax biochemically, yet its borderline position makes clinicoradiologic correlation essential, especially in the setting of hemorrhagic pleural fluid and recent complex surgery [9-11].

**Figure 8:**
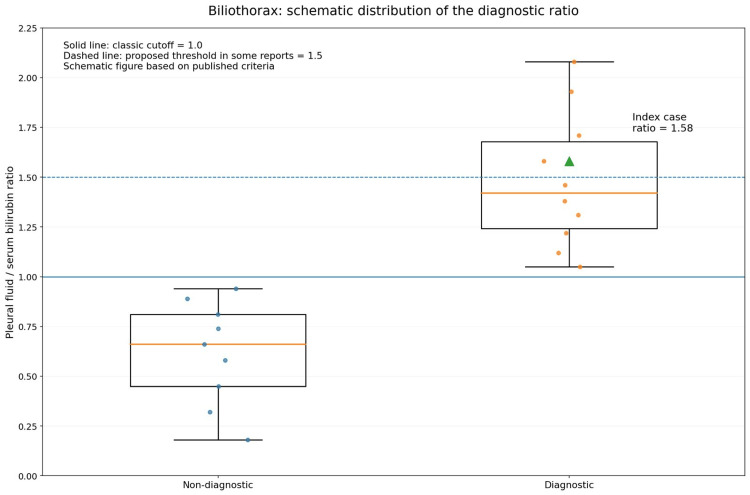
Schematic boxplot of the pleural fluid-to-serum bilirubin ratio in non-diagnostic pleural effusions and confirmed bilothorax The solid horizontal line marks the conventional diagnostic threshold of > 1.0, and the green triangle marker denotes the index case (ratio 1.58). This figure is intentionally schematic and literature-informed, designed to visualize the biochemical separation described in the current bilothorax literature rather than to represent pooled patient-level raw data.

Delayed post-traumatic diaphragmatic hernias, such as the one described in this case, are uncommon but potentially serious conditions. They may present with intestinal obstruction, ischemia, and the need for urgent surgical intervention [5,6]. Repair of the diaphragmatic defect and treatment of the compromised bowel are usually sufficient to control the associated inflammatory process. In this patient, the pleural effusion resolved following drainage and surgical management of the underlying cause, without the need for additional thoracic intervention.

Consequently, this case provides an important diagnostic insight: a pleural-to-serum bilirubin ratio > 1 should not automatically be assumed to represent true bilothorax. In the presence of hemorrhagic pleural fluid, predominance of indirect bilirubin, and absence of evidence of biliary leakage, pseudobilothorax due to intrapleural hemolysis should be considered [1,2].

## Conclusions

This report expands the differential diagnosis of pleural effusion in patients with post-traumatic diaphragmatic hernia and intestinal obstruction. Although a pleural-to-serum bilirubin ratio > 1 is considered a classic diagnostic criterion for bilothorax, in this case, the clinical context, the hemorrhagic appearance of the pleural fluid, and the predominance of indirect bilirubin suggest an alternative mechanism based on intrapleural hemolysis rather than a true biliary fistula.

Therefore, the diagnosis of bilothorax should not be established solely on the basis of an isolated biochemical value. Correlation with the clinical presentation, imaging findings, macroscopic appearance of the pleural fluid, and bilirubin fractions is essential. This case supports the concept of pseudobilothorax in the setting of a postoperative hemorrhagic pleural effusion following complicated diaphragmatic hernia repair.
